# Supplementary material for: Tombstone cost and longevity: The San Pedro Cemetery Museum in Medellín in Colombia
Source: PLoS One. 2024 Jan 19;19(1):e0293746. doi: 10.1371/journal.pone.0293746 (PMC10798633; doi:10.1371/journal.pone.0293746)
Supplement: S1 File — (PDF) [file pone.0293746.s002.pdf]

Tombstone cost and longevity: the San Pedro Cemetery Museum in Medellín in Colombia

Online Supplement

1. Preface

The purpose of this online supplement is to provide additional information about the methods used in our study, "Tombstone cost and longevity: the San Pedro Cemetery Museum in Medellín in Colombia." Section 2 describes the methods used to classify tombstone cost. Section 3 describes the mobile phone app used to facilitate data collection. It is important to remember that subjects in the present study were people who died in 2022, 2021, or 2020

2. Classification of tombstone cost

This section describes the step-by-step approach we used to classify tombstone cost. Section 2.1. describes how tombstone cost was scored according to its material. Section 2.2. explains how tombstone cost was scored according to its position. Section 2.3. describes how tombstone cost was scored according to its ornamentation. Section 2.4. explains how the scores were weighted. Section 2.5. describes how the overall classification was derived. Section 2.5. also includes a worked example.

2.1. Classification according to material

We visited several stonemasons near the San Pedro Cemetery Museum to determine the costs of the various materials used to make tombstones in the present day. Table S1 shows tombstone cost according to material. The high-cost category included red quartz, green Ubatuba granite from Brazil, and black marble. The high-cost category was given a score of three points. The medium-cost category included cream marble and grey marble. The medium-cost category was given a score of two points. The low-cost category included laminated stone and the plain stones provided by the cemetery free of charge. The low-cost category was given a score of one point. Costs are shown in Colombian pesos (COP). According to the World Bank, the exchange rate per US dollar (USD) was 3,743.59 in 2021. A red quartz tombstone might cost as much as COP 650,000 or USD 174.

2.2. Classification according to position in the gallery

Tombs are arranged in galleries in the San Pedro Cemetery Museum. Table S2 shows tombstone cost according to its position in the gallery. The high-cost category included tombs around head height that were easier to reach. The high-cost category was given a score of three points. The medium-cost category included tombs just above head height. The medium-cost category was given a score of two points. The low-cost category included tombs at the top of the gallery that were harder to reach and tombs at the bottom of the gallery that were harder to view. The low-cost category was given a score of one point. Tombs can be purchased, but most are rented. The cost of renting a tomb at head height is around COP 11,950,000, including the option of making a down payment of COP 5,975,000 and 24 monthly payments of COP 298,297. The cost of renting a tomb in a less desirable position is around COP 10,500,000, including the option of making a down payment of COP 5,250,000 and 24 monthly payments of COP 262,102 (costs do not sum because interest is charged on monthly payments).

2.3. Classification according to ornamentation

We visited several stonemasons near the San Pedro Cemetery Museum to determine the costs of the various ornamentations that might be added to a tombstone. Table S3 shows some of the ornamentations used in the present day. Costs varied from as little as COP 12,000 for a flowerpot to COP 150,000 for an engraving. The costs of the ornamentations on a tombstone were summed to create cost categories. The high-cost category included ornamentation to the sum of greater than COP 200,000. The medium-cost category included ornamentation to the sum of COP 101,000-200,000. The low-cost category included ornamentation to the sum of COP 0-100,000. The high-cost category was given a score of three points, the medium-cost category two points, and the low-cost category one point.

2.4. Weighting of tombstone characteristics

Tombstone cost was weighted by tombstone characteristics because differences in material had a greater influence on differences in cost than differences in position or differences in ornamentation. Tombstone material was given a weighting of 0.7, tombstone position was given a weighting of 0.1, and tombstone ornamentation was given a weighting of 0.2.

2.5. Final classification

Tombstone characteristics were multiplied by the respective weightings and summed to create the final score. For example, consider the tombstone in Figure 1. The tombstone material is cream marble, giving a score of 2 x 0.7 = 1.4. The tombstone position is head height, giving a score of 3 x 0.1 = 0.3. The tombstone has a flower box and an engraving and the price of these ornamentations is greater than COP 200,000, giving a score of 3 x 0.2 = 0.6. The final score is 1.4 + 0.3 + 0.6 = 2.3. Final scores of 2.4-3.0 were used to classify tombstones as high cost. Final scores of 1.7-2.3 were used to classify tombstones as medium cost. Final scores of 1.0-1.6 were used to classify tombstones as low cost. Therefore, the tombstone in Figure 1 is classified as medium cost.

3. Mobile phone app

Each death certificate held at the San Pedro Cemetery Museum had a code that indicated the location of the tomb in the cemetery. We created a mobile phone app to help us match up death certificates and tombstones. The app was created using Microsoft Power Apps. Table S4 shows the six steps used in collecting the data.

Table S1. Classification of tombstone cost according to material

Material	Example	Price (COP)	Cost category	Cost score
Red quartz	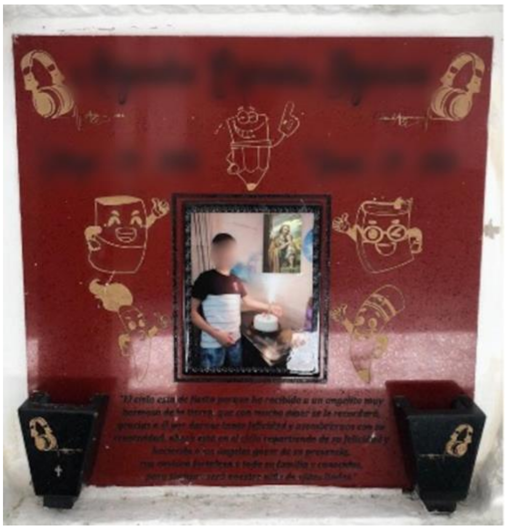	650,000	High	3
Green Ubatuba granite	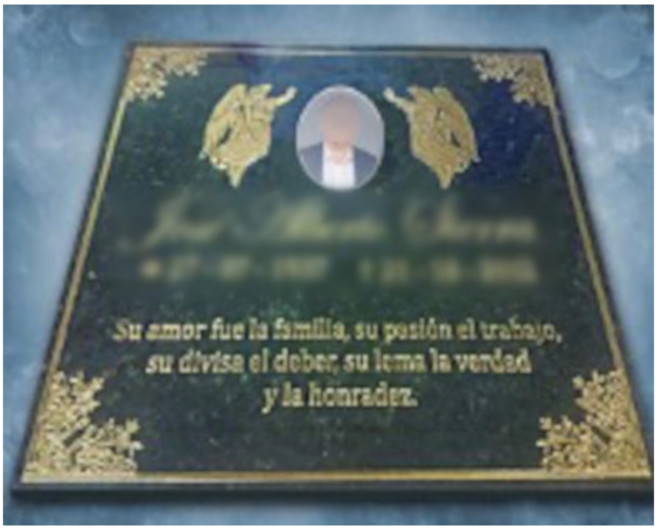	450,000	High	3
Black marble	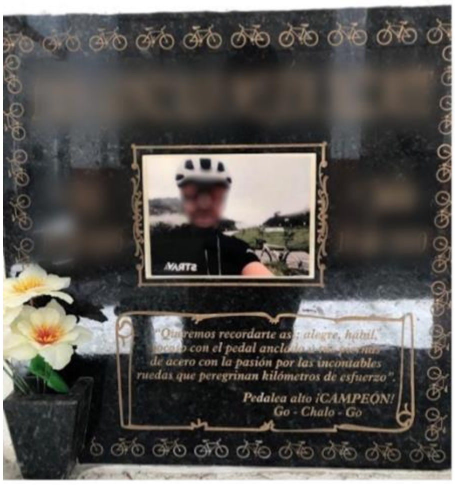	400,000	High	3

Table S1 (continued). Classification of tombstone cost according to material

Material	Example	Price (COP)	Cost category	Cost score
Cream marble	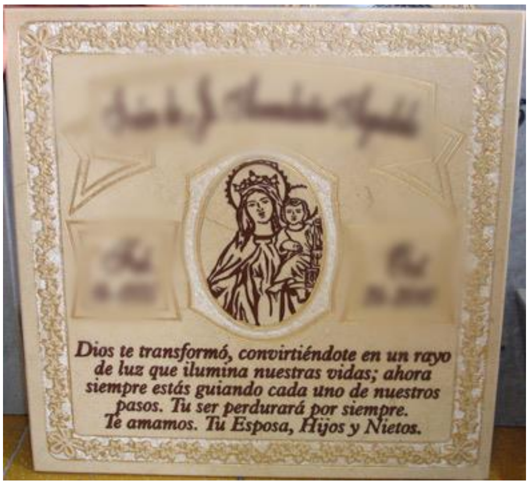	300,000	Medium	2
Grey marble	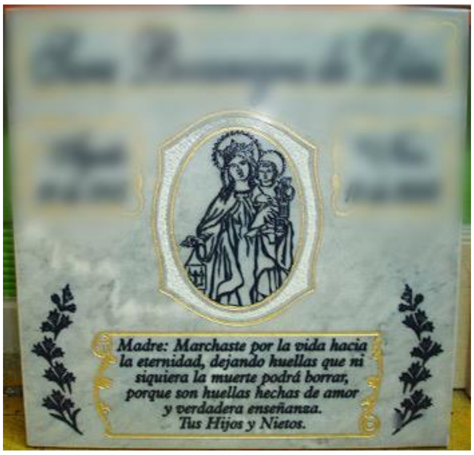	300,000	Medium	2
Laminated stone	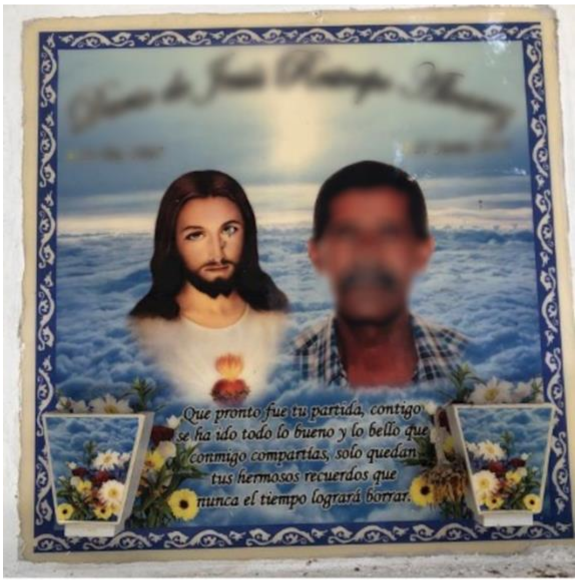	280,000	Low	1

Table S1 (continued). Classification of tombstone cost according to material

Material	Example	Price (COP)	Cost category	Cost score
Cemetery tombstone	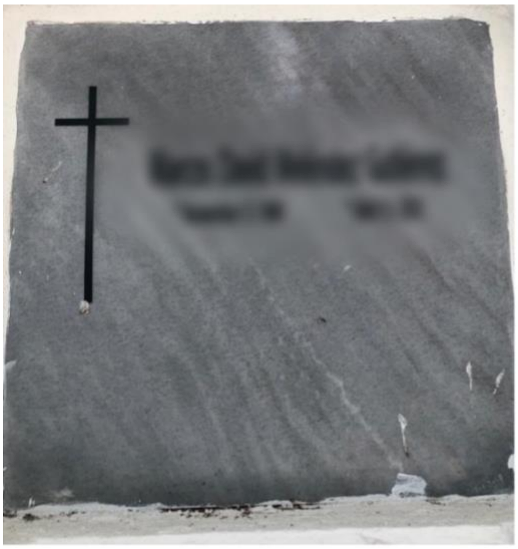	Free	Low	1
Cemetery tombstone with laminate	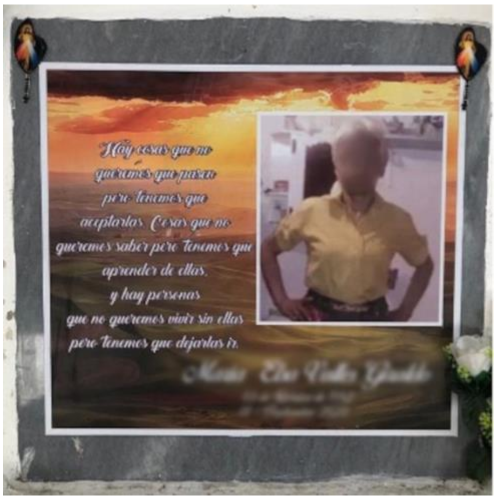	Free	Low	1

Table S2. Classification of tombstone cost according to its position in the gallery

Position in gallery	Cost category	Cost score
Row 6 (highest)	Low	1
Row 5	Low	1
Row 4	Medium	2
Row 3 (head height)	High	3
Row 2	High	3
Row 1 (lowest)	Low	1

Table S3. Ornamentations that may be added to a tombstone

Ornament	Example		Cost (COP)
Flowerpot	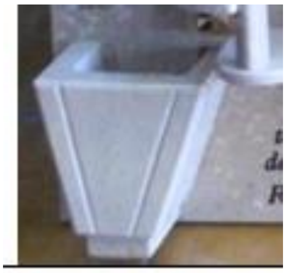		12,000-25,000
Photo of the deceased	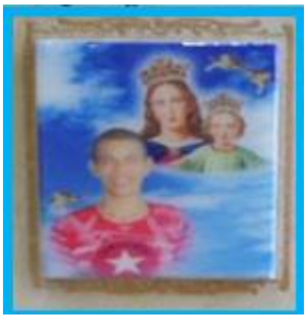		35,000-45,000
Engraving attached to the tombstone	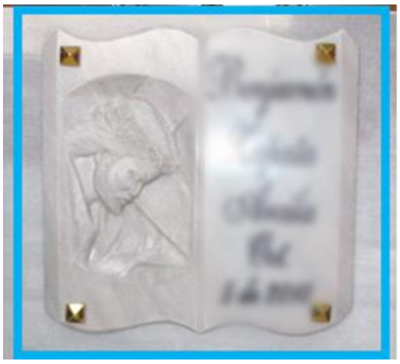		45,000
Flower box	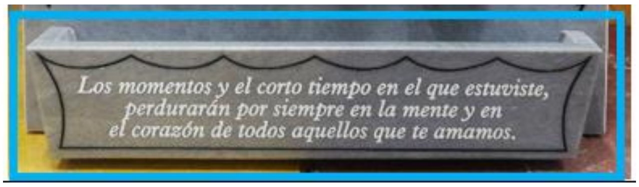		45,000-60,000
Balcony	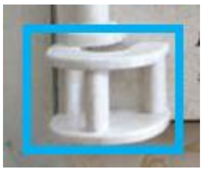	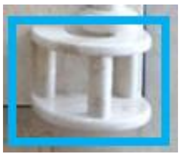	50,000
Figurine	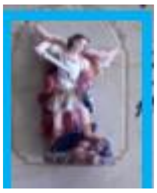		50,000

Table S3 (continued). Cost of ornaments added to tombstones

Ornament	Example	Cost (COP)
Engraving within the tombstone	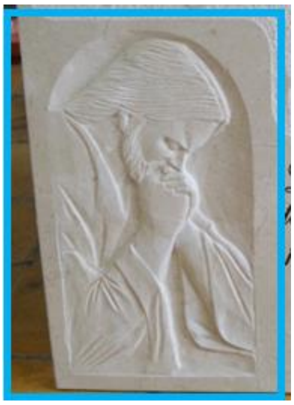	120,000-150,000
Roof	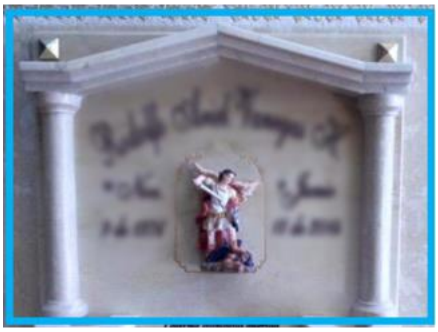	150,000-200,000

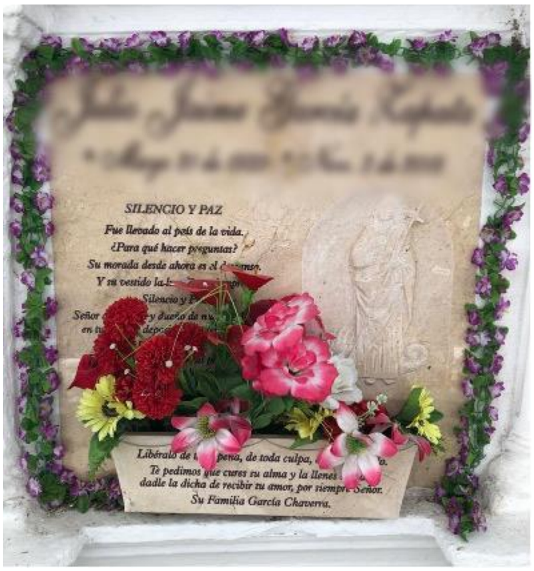

Figure S1. A tombstone in the San Pedro Cemetery Museum in Medellín in Colombia. The material is cream marble. The position of the tomb is head height. The ornamentations include a flower box and an engraving. The tombstone is classified as being of medium cost, as explained in Section 2.5.

Table S4. The six steps used in collecting data on the mobile phone app

Screenshot (in Spanish)	Step and description
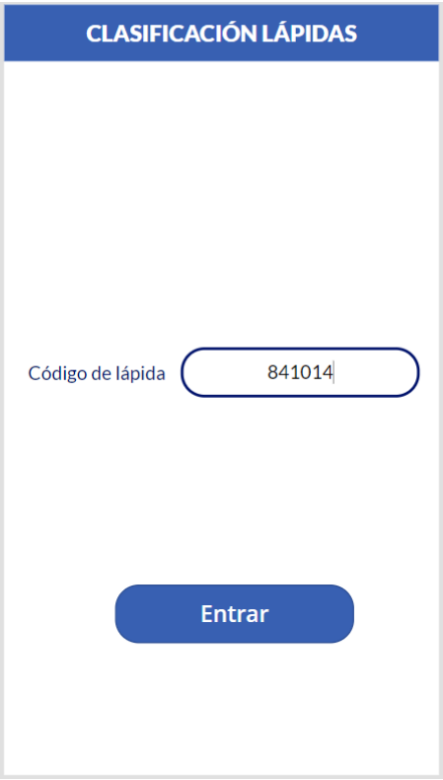	Step 1: Tombstone location. Each death certificate had a code that identified the location of the tomb in the cemetery. Here, the user is asked to enter the code.
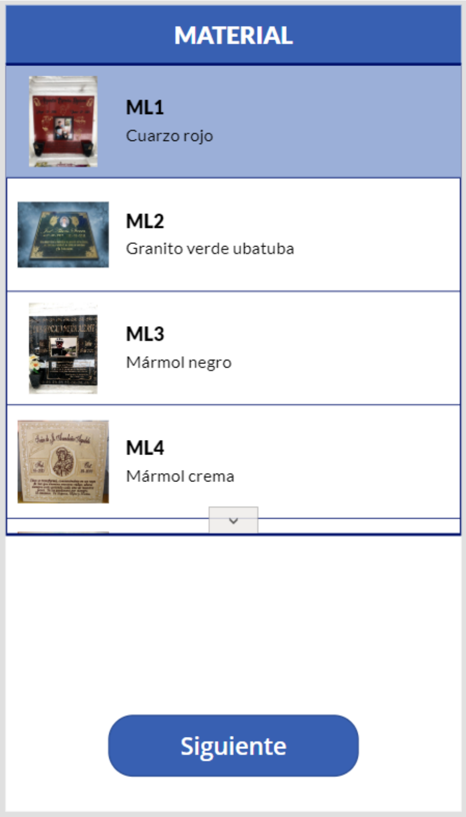	Step 2: Tombstone material. The user is shown eight examples of tombstone materials and is required to select the corresponding option.

Table S4 (continued). The six steps used in collecting data on the mobile phone app

Screenshot (in Spanish)	Step and description
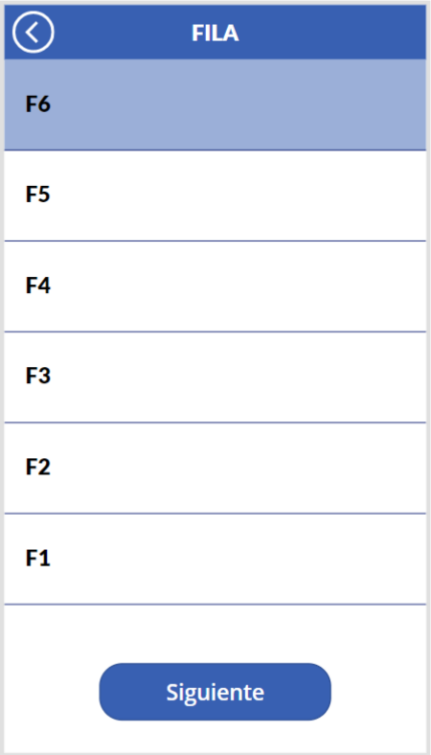	Step 3. Tombstone position. The user is shown six rows and is required to indicate the position of the tomb in the gallery.
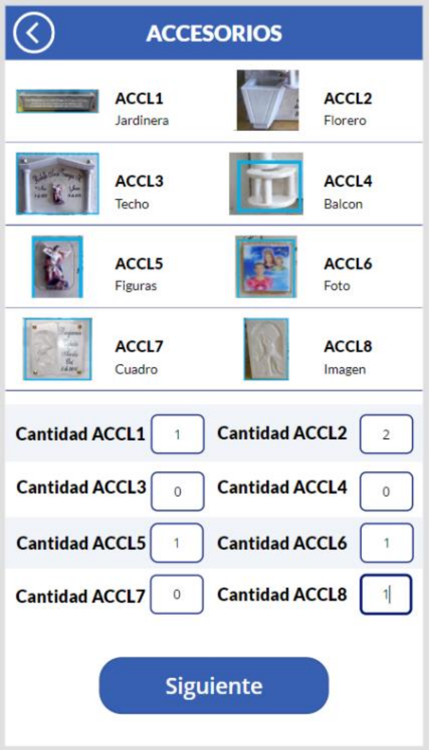	Step 4: Tombstone ornamentation. The user is shown examples of various ornaments that might be added to a tombstone and is required to enter the corresponding quantities.

Table S4 (continued). The six steps used in collecting data on the mobile phone app

Screenshot (in Spanish)	Step and description
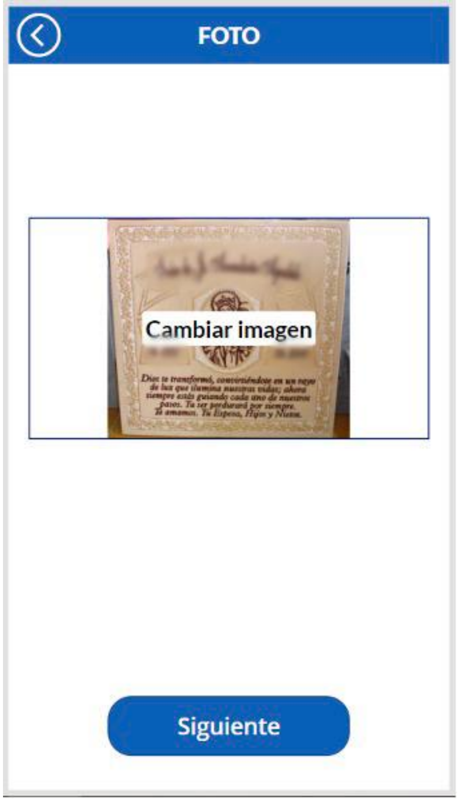	Step 5: Photo of the tombstone. The user must use the app to take a photo of the tombstone.
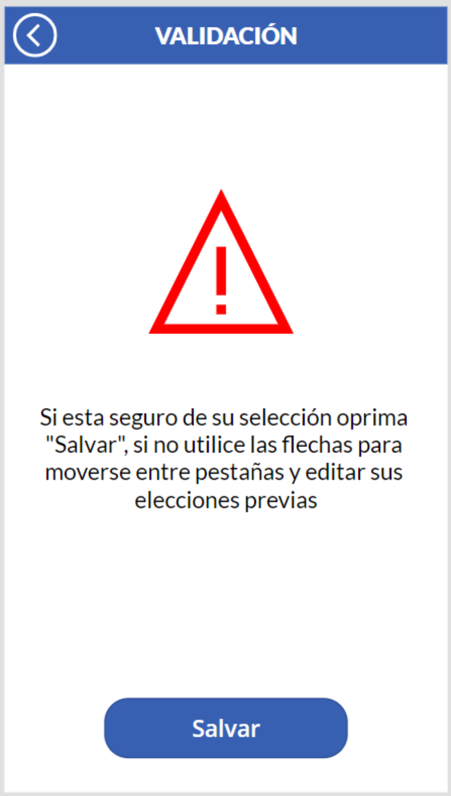	Step 6: Confirmation. The user is asked if they are sure about their selections and is invited to save their work. The data are stored securely in Microsoft OneDrive so that the investigators can resolve any doubts about tombstone material or tombstone ornamentation.
