# Supplementary material for: Tombstone cost and longevity: The San Pedro Cemetery Museum in Medellín in Colombia
Source: PLoS One. 2024 Jan 19;19(1):e0293746. doi: 10.1371/journal.pone.0293746 (PMC10798633; doi:10.1371/journal.pone.0293746)
Supplement: Attachment — Submitted filename: Response to reviewers.docx [file pone.0293746.s003.docx]

**Tombstone cost and longevity: the San Pedro Cemetery Museum in Medellín in Colombia**

**Response to reviewers**

*Reviewer comments*

[Comment 1] Please included in the manuscript the details of the ANCOVA model, the reason to use that approach, and the results in detail (table).

[Response 1] In the revised manuscript, it now says: “Analysis of variance was used in the main analyses because the dependent variable was continuous and the independent variable was categorical.” We have also added two tables. Table 2 is the analysis of variance summary table. Table 3 is the analysis of covariance summary table.

*Editor comments*

[Comment 1] Please include the results from your analysis controlling for sex, civil status, violent death, and year of death (mentioned in the manuscript but not reported in tables). In the methods, please proved further detail on the ANCOVA model and why you opted for that model.

[Response 1]. In the revised manuscript, we explain why we used analysis of variance: “Analysis of variance was used in the main analyses because the dependent variable was continuous and the independent variable was categorical.” We have also added detailed results of the analyses; please see Table 2 and Table 3.
