# Supplementary material for: Tombstone cost and longevity: The San Pedro Cemetery Museum in Medellín in Colombia
Source: PLoS One. 2024 Jan 19;19(1):e0293746. doi: 10.1371/journal.pone.0293746 (PMC10798633; doi:10.1371/journal.pone.0293746)
Supplement: Attachment — Submitted filename: Rev PLoS ONE.docx [file pone.0293746.s004.docx]

It is an interesting and novel study with data from a Latin American city. However, the manuscript lacks very relevant information about Medellin, the illegal activities that are generated from there. Some authors have classified Colombia as a case of a society with different behaviors from what is observed in other places, which is related to the drug trafficking and an armed conflict of several decades. Understanding Medellin society requires understanding the illegality associated with drug trafficking, a fact that may be changing the study's findings.

From the methodological point of view, why were the years 2020, 2021 and 2022 chosen? This must be very well supported, because it may be related to the findings. There is news and some academic publications suggesting that illicit activities did not stop during the pandemic. Injuries and deaths among young people are consequences in this city. For example, see:

Dellasega, Maria, and Judith Vorrath. "A Gangster’s Paradise?." Transnational Organised Crime in the Covid-19 Pandemic. SWP Comment, December 66 (2020).

Tamayo Gomez C. Organised Crime Governance in Times of Pandemic: The Impact of COVID-19 on Gangs and Drug Cartels in Colombia and Mexico. Bulletin of Latin American Research 2020;39:12–15.

Other publications that can contribute to the discussion are suggested:

Fernández-Niño J et al. The other social capital: a needed look at Latin America. Rev Panam Salud Publica 2014;35:291-2.

Rubio M. Perverse social capital – some evidence from Colombia. J Econ Issues. 1997;31:805–16.

Sanchez Parra T. What’s killing them: Violence beyond COVID-19 in Colombia. Crime Media Culture 2021, Vol. 17(1) 11–16.
