# Supplementary material for: Tombstone cost and longevity: The San Pedro Cemetery Museum in Medellín in Colombia
Source: PLoS One. 2024 Jan 19;19(1):e0293746. doi: 10.1371/journal.pone.0293746 (PMC10798633; doi:10.1371/journal.pone.0293746)
Supplement: Attachment — Submitted filename: Response to reviewers.docx [file pone.0293746.s005.docx]

**Tombstone cost and longevity: the San Pedro Cemetery Museum in Medellín in Colombia**

**Response to reviewers**

*Reviewer comments*

*Reviewer 1*

[Comment 1] It is an interesting and novel study with data from a Latin American city. However, the manuscript lacks very relevant information about Medellin, the illegal activities that are generated from there. Some authors have classified Colombia as a case of a society with different behaviors from what is observed in other places, which is related to the drug trafficking and an armed conflict of several decades. Understanding Medellin society requires understanding the illegality associated with drug trafficking, a fact that may be changing the study's findings.

[Response 1] Thank you for considering our study. We are glad that you found our work interesting and thought-provoking. All of the authors of the study have lived and worked in Medellín for many years and we agree with the reviewer: Medellín is a unique place! In the original manuscript, we did discuss gangs, drug dealing, and violence. We even mentioned that many people are destined to suffer a violent death: “Nacen con la lápida pegada”. We also cited the import work of the Colombian journalist, María Jimena Duzán (Duzán MJ. Sicarios. Crónicas que matan. Bogotá, Colombia: Penguin Random House; 2012). In the revised manuscript, we mention that the novelty of the study is one of its strengths and we cite the important work the reviewer mentioned: “Medellín may have been more affected by drug dealing and violence than any other city in the last 50 years [Duzán MJ. Sicarios. Crónicas que matan. Bogotá, Colombia: Penguin Random House; 2012; Dellasega M, Vorrath J. A Gangster’s Paradise? Transnational Organised Crime in the Covid-19 Pandemic 2020; Sanchez Parra T. What’s killing them: Violence beyond COVID-19 in Colombia. Crime, Media, Culture. 2020;17(1):11-6]; And, the present study offers a unique snapshot of life and death in Medellín in recent years.” We think that any further speculation about the inverse association between tombstone cost and longevity in Medellín is beyond the scope of this study.

[Comment 2] From the methodological point of view, why were the years 2020, 2021 and 2022 chosen? This must be very well supported, because it may be related to the findings. There is news and some academic publications suggesting that illicit activities did not stop during the pandemic. Injuries and deaths among young people are consequences in this city. For example, see: Dellasega, Maria, and Judith Vorrath. "A Gangster’s Paradise?." Transnational Organised Crime in the Covid-19 Pandemic. SWP Comment, December 66 (2020). Tamayo Gomez C. Organised Crime Governance in Times of Pandemic: The Impact of COVID-19 on Gangs and Drug Cartels in Colombia and Mexico. Bulletin of Latin American Research 2020;39:12–15.

[Response 2] In the methods section, we explain that most tombs are rented for no longer than three or four years: “The cemetery has more than 10,000 tombs for rent and the Ministry of Health stipulates that a body must remain interred for at least three years if the deceased was less than seven years old and for at least four years if the deceased was more than seven years old. After the minimum period of interment, the family can choose to transfer the body to another tomb or to cremate the body and store the ashes in a columbarium.” For this reason, we had to use the most recent death certificates.

[Comment 3] Other publications that can contribute to the discussion are suggested:

Fernández-Niño J et al. The other social capital: a needed look at Latin America. Rev Panam Salud Publica 2014;35:291-2. Rubio M. Perverse social capital – some evidence from Colombia. J Econ Issues. 1997;31:805–16. Sanchez Parra T. What’s killing them: Violence beyond COVID-19 in Colombia. Crime Media Culture 2021, Vol. 17(1) 11–16.

[Response 3]. We now cite the recent work of Sanchez Parra.

*Reviewer 2*

[Comment 1] I have gone through your manuscript and would like to raise a few points of clarification. Your hypothesis revolves around the relationship between age and tombstone cost. It might add depth to your study if you could perform the analysis segmented by different age groups. Perhaps you could consider delineating groups such as children, young adults, adults, and the elderly, and examine how the cost varies across these groups.

[Response 1]. Thank you for considering our manuscript. We appreciate your constructive and insightful comments. Our hypothesis is about the association between tombstone cost (exposure) and age at death (outcome). We cannot perform the stratification the reviewer suggests because, in stratification, one can only separate the sample into sub-samples according to exposures or confounders, not outcomes (Porta, M. A Dictionary of Epidemiology. Sixth Edition. Oxford: Oxford University Press; 2014).

It is also important to stress that the study was not designed to be divided into sub-samples, or strata. When a study is divided into strata, the efficiency (or precision) of the study can be affected dramatically (Rothman, K. J. and Lash, T. L. Precision and Study Size. In: Lash, T. et al., Modern Epidemiology. Fourth Edition Philadelphia: Wolters Kluwer; 2021). In other words, out study would have to be much bigger if it were to be divided into strata.

[Comment 2] It appears that you intended to estimate a certain sample size but the actual number of samples you obtained does not match this. Could you please clarify why you were unable to acquire the estimated number of samples?

[Response 2] We did indeed perform a sample size calculation to give us a rough idea of how many tombstones we might have to include. The estimated sample size was 1,528, including 699 tombstones of low cost, 699 of medium cost, and 130 of high cost. We actually created a sample of 2,273 tombstones before analyzing the data. We spent more than a year collecting the data and would have continued if the results had not been so clear.

[Comment 3] Your analysis controls for gender, but the sample size seems particularly small for high-cost male tombstones, with only 9 examples, and only one of these is under 36 years old. Could you comment on how this limited sample size might influence the robustness of your findings?

[Response 3] We would like to take this opportunity to clarify our approach to designing the study and analyzing the data. In the Methods section, we have added a paragraph explaining that the analyses were adjusted for potential confounders that might impact on our ability to draw valid conclusions. Please notice that potential confounders were chosen with reference to the literature. In the revised manuscript, it now says: “In observational studies, a confounding factor may be defined as a prognostic factor that has the potential to introduce material bias into the relationship between the exposure and the outcome [ROBINS-E Development Group. Risk Of Bias in Non-randomized Studies of Exposure (ROBINS – E). 2022. Available online: <https://www.riskofbias.info/welcome/robins-e-tool>]. Material bias is considered as bias sufficient to affect the direction of the estimated effect or to impact on the ability to draw valid conclusions about the relationship between the exposure (tombstone cost) and the outcome (longevity) [ROBINS -E Development Group, 2022]. Confounding factors that may introduce material bias into the relationship between tombstone cost and longevity include sex, civil status, violent death, and year of death [Kang-Auger et al., Eur J Epidemiol. 2021, 36, 1219-23; Hambleton et al., PLoS One, 2015, 10, e129778; Aburto et al., Sci Adv. 2023, 9, eadd9038]. Information about potential confounders was taken from the death certificates.” In the revised manuscript, we now use phrases like ‘adjusted for’ rather than phrases like ‘controlling for’.

[Comment 4] You also control for civil status in your study, yet this control variable seems inappropriate for some categories of your data, notably when considering children. How did you address this issue within your analysis?

[Response 4] This is a very interesting comment. We agree that the missing data could cause bias and we have added a sensitivity analysis: “Civil status was missing from 514 subjects, which could cause bias if the missing subjects were children or young adults. However, the results were not changed when missing values were coded as not married or with partner [age at death was 61.8 years (60.9, 62.7) in the low-cost group, 61.1 years (59.4, 62.9) in the medium-cost group, and 45.6 years (40.0, 51.3) in the high-cost group].

[Comment 5] Finally, your claim that "the glorification of violence may help explain why the most expensive tombstones were those of the young" raises a question. How do you reconcile this statement with your methodology, specifically when you are controlling for violent deaths? How does this control affect your conclusions?

[Response 5] We hope that, in the revised manuscript, it is now clear that we wished to adjust for potential confounders, rather than control for them per se. We find it reassuring that the results of the ANOVA and ANCOVA analyses are similar because it suggests that the potential confounders did not have a great impact on the association between tombstone cost and longevity.
