# Supplementary material for: Tombstone cost and longevity: The San Pedro Cemetery Museum in Medellín in Colombia
Source: PLoS One. 2024 Jan 19;19(1):e0293746. doi: 10.1371/journal.pone.0293746 (PMC10798633; doi:10.1371/journal.pone.0293746)
Supplement: Attachment — Submitted filename: Response to Reviewers.docx [file pone.0293746.s006.docx]

**Tombstone cost and longevity: the San Pedro Cemetery Museum in Medellín in Colombia**

*Response to reviewers*

*Reviewer comments*

*Reviewer #1*

[Comment 1] The new version is very similar to the previous one. I suggest that authors not only analyze data but postulate explanations for what they observe. There are sociological and anthropological studies that could help explain the findings. This is important to guide future similar research. I hope they do not give up on theoretically deepening the results obtained.

[Response 1] In the Discussion section in the revised manuscript, we have added a paragraph about Medellín that should help the reader place the results of the study in context. Please see below. We do not want to label or stigmatize the poor and the marginalized and we reiterate that we have no way of knowing whether the young subjects in the present study were victims of gang-related crime.

“Medellín changed from being a small town to a city in the 1970’s (Museo Casa de la Memoria, 2023). The population of Medellín doubled between 1965 and 1985 because of three waves of migrants: families fleeing political violence in the countryside; people looking to work and study and people looking for a better quality of life; and, people attracted by the government’s promotion of urban developments (Museo Casa de la Memoria, 2023). The authorities struggled to cope with the growing population and there was an industrial crisis, high unemployment, and a large black market controlled by drug-traffickers (Museo Casa de la Memoria, 2023). Medellín was characterized by two phenomena of urban growth in the 1970’s: there was planned and regulated urbanization along the west side of the River Medellín and there was informal and illegal development on the hillsides of the north of the city (Museo Casa de la Memoria, 2023). More than 57,000 young people were murdered in Medellín between 1979 and 2018 and people aged 18 to 24 years are still the main victims of murder (Alcaldía de Medellín, 2023). This long cycle of violence has led many academics to conclude that the poor and the marginalized are destined to join a gang and die young (Alcaldía de Medellín, 2019). Murder is the ultimate manifestation of taking the law into one’s own hands in poor and marginalized communities in Medellín and elsewhere in Latin America (Alcaldía de Medellín, 2019) and an investigation of 12 young murder victims found that 11 of them had experienced threats and violent situations weeks, months, and even years before their deaths (Casa de las Estrategias, 2017). It is the precarious nature of many young people’s lives in Medellín that leads to violence (Alcaldía de Medellín, 2019). Precarious economic conditions mean that many young people have limited access to essential goods and services (Alcaldía de Medellín, 2019). Precarious social conditions mean that many young people do not enjoy the same social fabric and the same social mobility as others (Alcaldía de Medellín, 2019). Precarious cultural conditions mean that many young people lack appropriate role models and are exposed to perverse norms and values (Alcaldía de Medellín, 2019). Precarious access to formal justice means that violence is often used to settle disputes (Alcaldía de Medellín, 2019). More than 200 young people in Medellín took part in workshops and semi-structured interviews between 2018 and 2019, including young people who had committed murder (Alcaldía de Medellín, 2019). Nine external factors were found to be associated with violent crime and murder: selling and consuming narcotic drugs; the need or desire to earn money; dropping out of school; displacement by violence; coming from a violent family or violent community; living in a community run by gangs; ‘invisible borders’ and limited mobility between communities; the opportunity to join a local gang; and, the exposure to uncontrolled violence (Alcaldía de Medellín, 2019). Four profiles of young people at risk of being the victim or the perpetrator of murder were also identified: members of criminal gangs; members of less formal groups of drug users and petty criminals; independent criminals who are not affiliated with a gang; and, young people outside of the criminal world who kill after taking drugs or getting into an argument (Alcaldía de Medellín, 2019). Gang-related crime was the main cause of murder in young people in Medellín in 2020, 2021, and 2022 (Alcaldía de Medellín, 2023), but we have no way of knowing whether the young subjects in the present study were victims of gang-related crime.”

**References**

Alcaldía de Medellín. (2019). Factores que inciden en el homicidio de jóvenes en Medellín: Propuesta de acción en clave de gobernanza colaborativa. Retrieved from <https://www.medellin.gov.co/es/wp-content/uploads/2022/10/factores-que-inciden-en-el-homicidio-de-jovenes.pdf>

Alcaldía de Medellín. (2023). Sistema de Información para la Seguridad y la Convivencia (SISC). Retrieved from <https://www.medellin.gov.co/es/secretaria-seguridad/sisc/>

Casa de las Estrategias. (2017). Los jóvenes destinados al homicidio en Medellín. In J. Giraldo (Ed.), *Territorios y sociabilidades violentas. Santo Domingo, San Juan, Sao Paulo, Cali y Medellín* (pp. 177-197). Medellín: Universidad EAFIT.

Museo Casa de la Memoria. (2023). Década de los 70. Retrieved from <https://www.museocasadelamemoria.gov.co/medellin708090/decada-del-70/>
